# Development and validation of a preoperative nomogram for predicting survival of patients with locally advanced prostate cancer after radical prostatectomy

**DOI:** 10.1186/s12885-020-6565-5

**Published:** 2020-02-04

**Authors:** Xianghong Zhou, Qingyang Ning, Kun Jin, Tao Zhang, Xuelei Ma

**Affiliations:** 1 Department of Biotherapy, West China Hospital and State Key Laboratory of Biotherapy, Sichuan University, Chengdu, People’s Republic of China; 2 Department of Urology, Institute of Urology and National Clinical Research Center for Geriatrics and Center of Biomedical Big Data, West China Hospital, Sichuan University, Chengdu, Sichuan Province People’s Republic of China; 3 West China School of Medicine, Sichuan University, Chengdu, People’s Republic of China

**Keywords:** Prostate cancer, Radical prostatectomy, Nomogram

## Abstract

**Background:**

For selected locally advanced prostate cancer (PCa) patients, radical prostatectomy (RP) is one of the first-line treatments. We aimed to develop a preoperative nomogram to identify what kinds of patients can get the most survival benefits after RP.

**Methods:**

We conducted analyses with data from the Surveillance, Epidemiology, and End Results (SEER) database. Covariates used for analyses included age at diagnosis, marital status, race, American Joint Committee on Cancer (AJCC) 7th TNM stage, Prostate specific antigen, Gleason biopsy score (GS), percent of positive cores. We estimated the cumulative incidence function for cause-specific death. The Fine and Gray’s proportional subdistribution hazard approach was used to perform multivariable competing risk analyses and reveal prognostic factors. A nomogram was built by these factors (including GS, percent of positive cores and N stage) and validated by concordance index and calibration curves**.** Risk stratification was established based on the nomogram.

**Results:**

We studied 14,185 patients. N stage, GS, and percent of positive cores were the independent prognostic factors used to construct the nomogram. For validating, in the training cohort, the C-index was 0.779 (95% CI 0.736–0.822), and in the validation cohort, the C-index was 0.773 (95% CI 0.710–0.836). Calibration curves showed that the predicted survival and actual survival were very close. The nomogram performed better over the AJCC staging system (C-index 0.779 versus 0.764 for training cohort, and 0.773 versus 0.744 for validation cohort). The new stratification of risk groups based on the nomogram also showed better discrimination than the AJCC staging system.

**Conclusions:**

The preoperative nomogram can provide favorable prognosis stratification ability to help clinicians identify patients who are suitable for surgery.

## Background

Prostate cancer (PCa) is one of the most common malignant tumors of males around the world, and it is estimated that there will be 174,650 new male cases in the United States in 2019 [1]. The European Association of Urology defined PCa with cT3–4 or N+ as locally advanced PCa [2]. Patients with locally advanced PCa have an increased risk of disease progression and cancer-specific death [2]. Due to many factors like the American national guidelines advising against PSA screening, increasing applications for active surveillance, etc., the proportion of men diagnosed with locally advanced PCa has increased [3, 4]. Nowadays, the standard treatment of locally advanced PCa is still unclear due to the absence of level 1 evidence. For selected locally advanced PCa patients, radical prostatectomy (RP) is one of the first-line treatments recommended by the guidelines [2, 5]. However, it is still controversial as to which types of patients could get the most survival benefits from RP.

Some studies about the risk stratification of patients with high-risk PCa (including locally advanced PCa) after RP had been reported. Previous researchers performed a retrospective analysis of 315 high-risk PCa patients after RP in their hospital. They selected biochemical progression as the primary endpoint, and reported that the risk of biochemical progression of high-risk PCa after RP could be stratified by Gleason Score (GS) at biopsy (≥ 8) and % positive core (≥ 30%) [6]. Another research team studied 813 high-risk patients undergoing RP, and found 3 preoperative criteria including stage cT2 or greater, prostate specific antigen (PSA) > 20 ng/mL, and GS > 8. In their study, the number of meeting preoperative criteria was significantly predictive for recurrence-free survival (RFS), and overall survival (OS) [7]. Although these studies had proposed their own risk stratification models for high-risk PCa patients undergone RP, the patients involved in these studies were not fully compliant with the current more common definition of locally advanced PCa, the impact of prognostic factors on patient survival outcome was not quantitative, the weight between the various prognostic factors was not clear enough and the cohorts were not large enough.

To circumvent these defects, we evaluated the prostate cancer-specific survival (CSS) at a large cohort to assess potential preoperative prognostic factors, and developed a preoperative nomogram. This nomogram could be used to predict the CSS of patients with locally advanced PCa after RP and to identify what kinds of patients can get the most survival benefits from RP.

## Methods

### Patient selection

The Surveillance, Epidemiology, and End Results (SEER) database is a free dataset made up of 18 population-based cancer registries. It releases cancer patients’ general information annually and has almost covered 25% population of the United States [8].

**Fig. 1:**
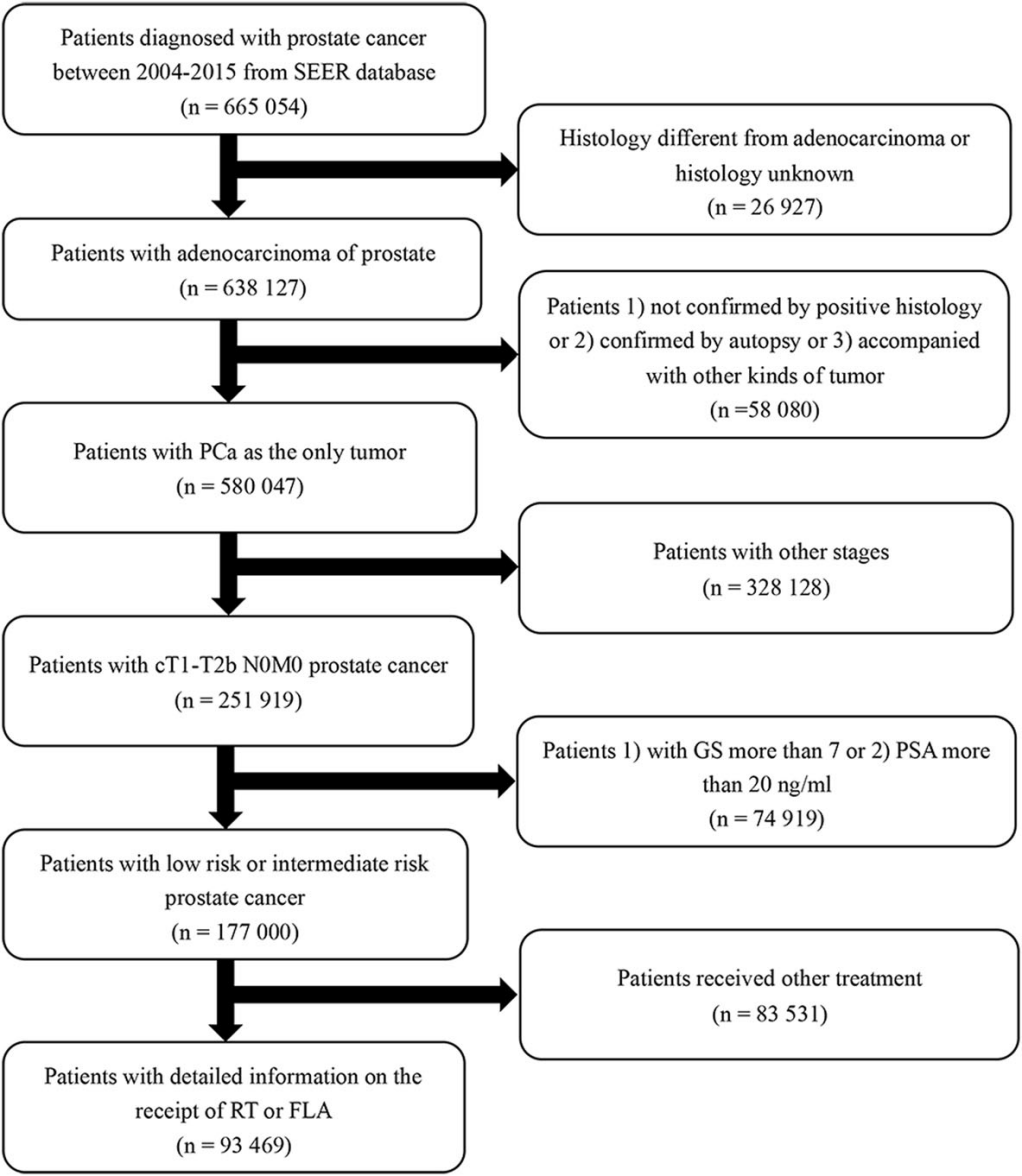
Flowchart describing the selection of patients in the Surveillance, Epidemiology, and End Results (SEER) database, 2010–2016

From the SEER database, patients with a diagnosis of adenocarcinoma of the prostate (International Classification of diseases-O-3 code: C61.9) between 2010 and 2016 were selected. The TNM stages were assessed by the 7th edition of American Joint Committee on Cancer [AJCC] Cancer Staging Manual [9]. Inclusion and exclusion criteria were shown in the flowchart in detail (Fig. 1). All the included patients were randomly divided into the training cohort and validation cohort with a ratio of 7:3.

### Variables and endpoint

Data for each patient were extracted from the SEER database including age at diagnosis, marital status, race, AJCC 7th TNM stage, PSA, Gleason biopsy score (GS), percent of positive cores at biopsy (% positive core), and follow-up information. Age was categorized as ≤49 years, 50–59 years, 60–69 years, and ≥ 70 years. PSA was categorized as ≤10 ng/mL, 10–20 ng/mL (not including 10), and > 20 ng/mL. GS was classified as ≤6, 7(3 + 4), 7(4 + 3), 8, and ≥ 9. % positive core was classified as 0–25, 25–50% (not including 25%), 50–75% (not including 50%), and 75–100% (not including 75%). In addition to follow-up information, all clinical factors were obtained before undergoing RP. Due to the limitation of the SEER database, the information about detail procedures used to determine the lymph node positive was not been provided [10].

CSS was used as the primary endpoint. CSS was measured by all deaths caused by prostate cancer, complications of treatments, or unknown processes in patients with active prostate cancer. Follow-up time was defined as the time between the first treatment and the patient’s death or last follow-up.

### Statistical analysis

A χ ^2^ test was used to expose the difference between the training cohort and validation cohort in categorical variables, and the results were presented as the frequency with its proportion. Kaplan–Meier method and log-rank test were used to expose each potential prognostic variable. Variables in the univariate analysis with *p*-value < 0.05 were chosen for multivariate Cox proportional hazard regression to identify the independent prognostic factors. In addition, many patients with locally advanced PCa are the elderly with many comorbidities. Thus, there is a high risk of non-cancer competing risk events leading to death in the long-term survival [11]. In order to make the research more precise, we further implemented competing risk analyses. We estimated the cumulative incidence function (CIF) for cause-specific death, and tested the differences by Gray’s test to expose each prognostic variable. The Fine and Gray’s proportional subdistribution hazard approach was used to conduct competing risk multivariable analyses. Based on clinical significance and *p*-value < 0.05 in competing risk multivariable analyses, these independent prognostic factors were selected to construct a nomogram to predict 5-year cancer-specific mortality (CSM) probabilities of patients.

The performance of the nomogram was validated by measuring discrimination and calibration in both training set and validation set. The discrimination of the nomogram was assessed by C-index (concordance index). The value of C-index ranged from 0.5 (no discrimination) to 1 (perfect discrimination), and higher C-index showed better discrimination of the prognostic model [12]. By comparing the plot of predicted probabilities from the nomogram with the plot of actual probabilities of CSS, the calibration curve was used to compare the predicted survival using nomogram with the actual observed survival, with 1000 resamples of bootstrapping. In addition, we conducted a risk stratification of the nomogram total risk score. The cut-off values were determined using X-tile software and by the minimal *p*-value approach [13]. The discrimination abilities between the nomogram and AJCC staging system were compared by C-index and Kaplan–Meier curve.

The statistical software packages R (The R Foundation) and R packages cmprsk, rms, survival, mstate, and regplot were used in the above statistical analyses. A *p*-value < 0.05 was considered statistically significant.

## Results

### Patient characteristics

A total of 14,185 eligible patients were included in the current analysis as the primary cohort. Among all patients, 9932 patients were placed within the training cohort, and 4253 patients were placed within the validation cohort randomly. Detail patients’ characteristics were shown in Table 1. The median follow-up was 43 months (95% CI 42–44 months). There were no statistically significant differences in baseline characteristics between the training cohort and the validation cohort.

**Table 1:** Descriptive characteristics of 14,185 locally advanced prostate cancer patients undergoing radical prostatectomy between 2010 and 2016 from the Surveillance Epidemiology and End Results database. Primary 14,185 patients were randomly divided into 2 cohorts: training cohort and validation cohort

Variable	Primary Cohort (*n* = 14,185)		Training Cohort (*n* = 9932)		Validation Cohort (*n* = 4253)		*P*-value
	Number	%	Number	%	Number	%	
Age							0.131
< 50	514	3.6	356	3.6	158	3.7	
50–59	4092	28.9	2907	29.3	1185	27.9	
60–69	7460	52.6	5163	52.0	2297	54.0	
> 69	2119	14.9	1506	15.2	613	14.4	
Race							0.658
White	11,445	80.7	8033	80.9	3412	80.2	
Black	1852	13.0	1282	12.9	570	13.4	
Other^a^	888	6.3	617	6.2	271	6.4	
Marital Status							0.501
Married	10,429	73.5	7289	73.4	3140	73.8	
Single^b^	3001	21.2	2100	21.1	901	21.2	
Unknown	755	5.3	543	5.5	212	5.0	
T stage							0.019
T1–2	290	2.0	185	1.9	105	2.5	
T3–4	13,895	98.0	9747	98.1	4148	97.5	
N stage							0.077
N0	12,215	86.1	8586	86.4	3629	85.3	
N1	1970	13.9	1346	13.6	624	14.7	
PSA level (ng/ml)							0.012
≤ 10	9447	66.6	6682	67.3	2765	65.0	
10–20	3043	21.5	2066	20.8	977	23.0	
> 20	1695	11.9	1184	11.9	511	12.0	
GS biopsy							0.402
≤ 6	2142	15.1	1510	15.2	632	14.9	
7 (3 + 4)	4571	32.2	3234	32.6	1337	31.4	
7 (4 + 3)	3015	21.3	2080	20.9	935	22.0	
8	2436	17.2	1684	17.0	752	17.7	
≥ 9	2021	14.2	1424	14.3	597	14.0	
% positive core biopsy							0.638
00–25%	2986	21.1	2088	21.0	898	21.1	
25–50%	4982	35.1	3515	35.4	1467	34.5	
50–75%	3008	21.2	2081	21.0	927	21.8	
75–100%	3209	22.6	2248	22.6	961	22.6	
AJCC staging system							0.140
IIIB	10,820	76.3	7591	76.4	3229	75.9	
IIIC	1395	9.8	995	10.0	400	9.4	
IVA	1970	13.9	1346	13.6	624	14.7	

*Abbreviation*: *AJCC* American Joint Committee on Cancer

^a^Other: American Indian/AK Native, Asian/Pacific Islander

^b^Single: Divorced, Separated, Single (never married), Widowed, unmarried

### Determination of independent preoperative prognostic factors

The Fine and Gray’s proportional subdistribution hazard approach was performed to demonstrate the independent preoperative prognostic factors in the training cohort. Table 2 showed the detail univariate and multivariate analysis results. In the univariate analyses, age, N stage, PSA, GS, % positive core biopsy were variables with significant impact on CSS.

**Table 2:** Univariate analyses, multivariate analyses of preoperative prognostic factors influencing cancer-specific survival outcomes in the training cohort

Variable	Univariate analyses (KM) *p*-value	Univariate analyses (CIF) *p*-value	Multivariate Cox regression analyses HR (95%CI)	*P*-value	Multivariate Competing risk analyses sdHR (95%CI)	*P*-value
Age	0.041	0.047				
< 50			Ref.		Ref.	
50–59			1.111 (0.440–2.804)	0.824	1.110 (0.440–2.802)	0.826
60–69			0.805 (0.321–2.0203)	0.645	0.803 (0.320–2.015)	0.641
> 69			1.291 (0.495–3.373)	0.602	1.284 (0.492–3.355)	0.609
Race	0.860	0.852				
White			Ref.		Ref.	
Black			0.988 (0.587–1.661)	0.962	0.987 (0.587–1.661)	0.962
Other			0.961 (0.487–1.898)	0.908	0.960 (0.486–1.897)	0.907
Marital Status	0.790	0.815				
Married			Ref.		Ref.	
Single			1.004 (0.675–1.495)	0.984	1.003 (0.674–1.493)	0.988
Unknown			1.193 (0.602–2.364)	0.613	1.189 (0.600–2.357)	0.619
T stage	0.300	0.304				
T1–2			Ref.		Ref.	
T3–4			6.511 (0.897–47.269)	0.064	6.493 (0.895–47.142)	0.064
N stage	< 0.001	< 0.001				
N0			Ref.		Ref.	
N1			2.431 (1.695–3.489)	< 0.001	2.429 (1.693–3.483)	< 0.001
PSA level (ng/ml)	< 0.001	< 0.001				
≤ 10			Ref.		Ref.	
10–20			1.141 (0.765–1.702)	0.519	1.140 (0.764–1.701)	0.521
> 20			1.472 (0.962–2.253)	0.075	1.473 (0.962–2.253)	0.075
GS biopsy	< 0.001	< 0.001				
≤ 6			Ref.		Ref.	
7 (3 + 4)			4.252 (1.277–14.152)	0.018	4.253 (1.2779–14.157)	0,018
7 (4 + 3)			6.096 (1.827–20.338)	0.003	6.099 (1.828–20.349)	0.003
8			9.796 (2.972–32.286)	< 0.001	9.8068 (2.975–32.322)	< 0.001
≥ 9			18.879 (5..88–61.576)	< 0.001	18.888 (5.791–61.605)	< 0.001
% positive core biopsy	< 0.001	< 0.001				
00–25%			Ref.		Ref.	
25–50%			1.099 (0.599–2.018)	0.760	1.100 (0.599–2.020)	0.758
50–75%			1.145 (0.604–2.173)	0.678	1.145 (0.604–2.173)	0.678
75–100%			2.176 (1.219–3.8837)	0.009	2.174 (1.218–3.881)	0.009

*Abbreviation*: *KM* Kaplan-Meier method, *CIF* Cumulative incidence function, *sdHR* Subdistribution hazard ratio, *Ref.* Reference, *% positive core biopsy* Percent of positive cores at biopsy

In the Fine and Gray’s proportional subdistribution hazard approach, N stage, GS, % positive core biopsy remained significantly, while age and PSA did not show significant impact on CSS. These significant variables were thought as independent prognostic factors of CSS for locally advanced PCa patients after RP.

### Construction and validation of the nomogram

**Fig. 2:**
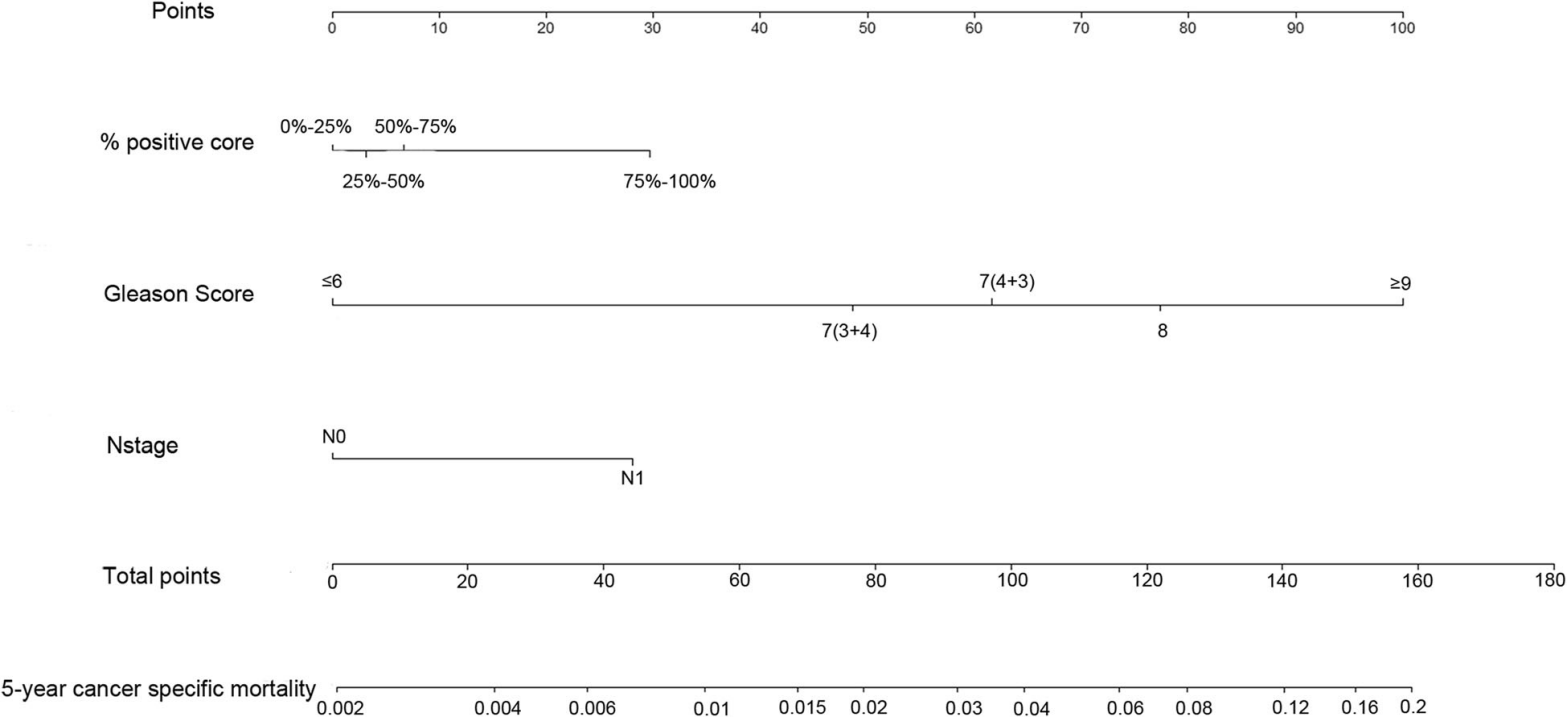
Nomogram for cancer specific mortality (CSM) at 5 years in locally advanced prostate cancer patients after undergoing surgery. Abbreviations: % positive core, percent of positive cores in total cores at biopsy

Using the independent prognostic factors including N stage, GS, and %positive core biopsy, a nomogram was constructed to predict each locally advanced PCa patient’s probability of CSM at 5 years after RP (Fig. 2).

**Fig. 3:**
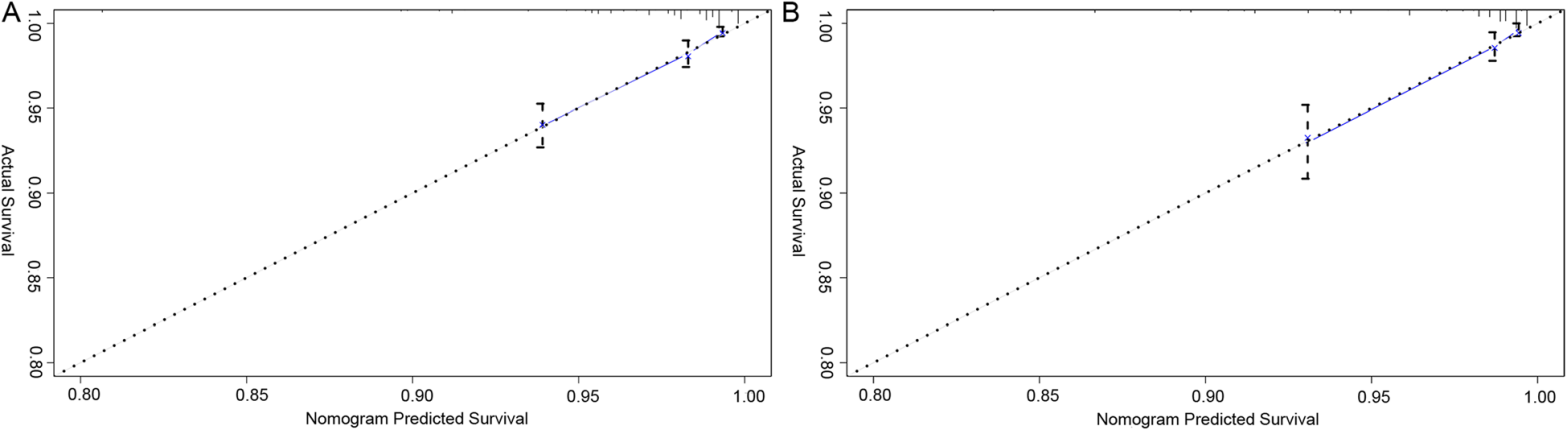
Calibration curves for cancer specific survival (CSS) at 5 years in locally advanced prostate cancer patients after undergoing surgery in the training cohort (**a**) and the validation cohort (**b**). The horizontal axis is the survival rate predicted by the nomogram, and the vertical axis is the actual survival rate. The dashed line indicates the predicting survival rate completely fits the actual survival rate

The nomograms were validated using C-index and calibration curve. In the training cohort, the C-index was 0.779 (95% CI 0.736–0.822). In the validation cohort, the C-index was 0.773 (95% CI 0.710–0.836). The C-index values indicated good discrimination of the nomogram (> 0.7). The calibration curves showed good agreement between prediction by nomogram and actual 5-year CSS in both training cohort and validation cohort (Fig. 3).

### Definition of nomogram risk group stratification

The score corresponding to each nomogram variable was listed in detail in Table 3. By summing the score for each nomogram variable, we got the nomogram total risk score for each patient in both the training cohort and the validation cohort. The patients were divided into three nomogram risk groups for CSS: a low-risk group with 0–77 points, a middle-risk group with 79–108 points, a high-risk group with no less than 112 points.

**Table 3:** Detailed risk scores of all prognostic factors in the nomogram

Variables	Nomogram risk score
N stage	
N0	0
N1	28
Gleason Score	
≤ 6	0
7 (3 + 4)	49
7 (4 + 3)	62
8	77
≥ 9	100
Percent of positive cores	
0–25%	0
25–50%	3
50–75%	7
75–100%	30
Total points	Predicted probability of 5-year CSM at 5 years
0	0.20%
40	0.64%
80	2.10%
120	6.75%
160	20.52%

*Abbreviation*: *CSM* Cancer specific mortality

### Comparison of nomogram with AJCC staging system

We compared the nomogram with the 8th edition AJCC staging system. In the training cohort, the C-index of AJCC staging system was 0.764 (95% CI 0.719–0.809), and the C-index of the nomogram was 0.779 (95% CI 0.736–0.822). In the validation cohort, the C-index of AJCC staging system was 0.744 (95% CI 0.707–0.817), and the C-index of the nomogram was 0.773 (95% CI 0.710–0.836). In both cohorts, the nomogram performed better in C-index, indicating that the nomogram had a better discrimination ability than AJCC staging system.

**Fig. 4:**
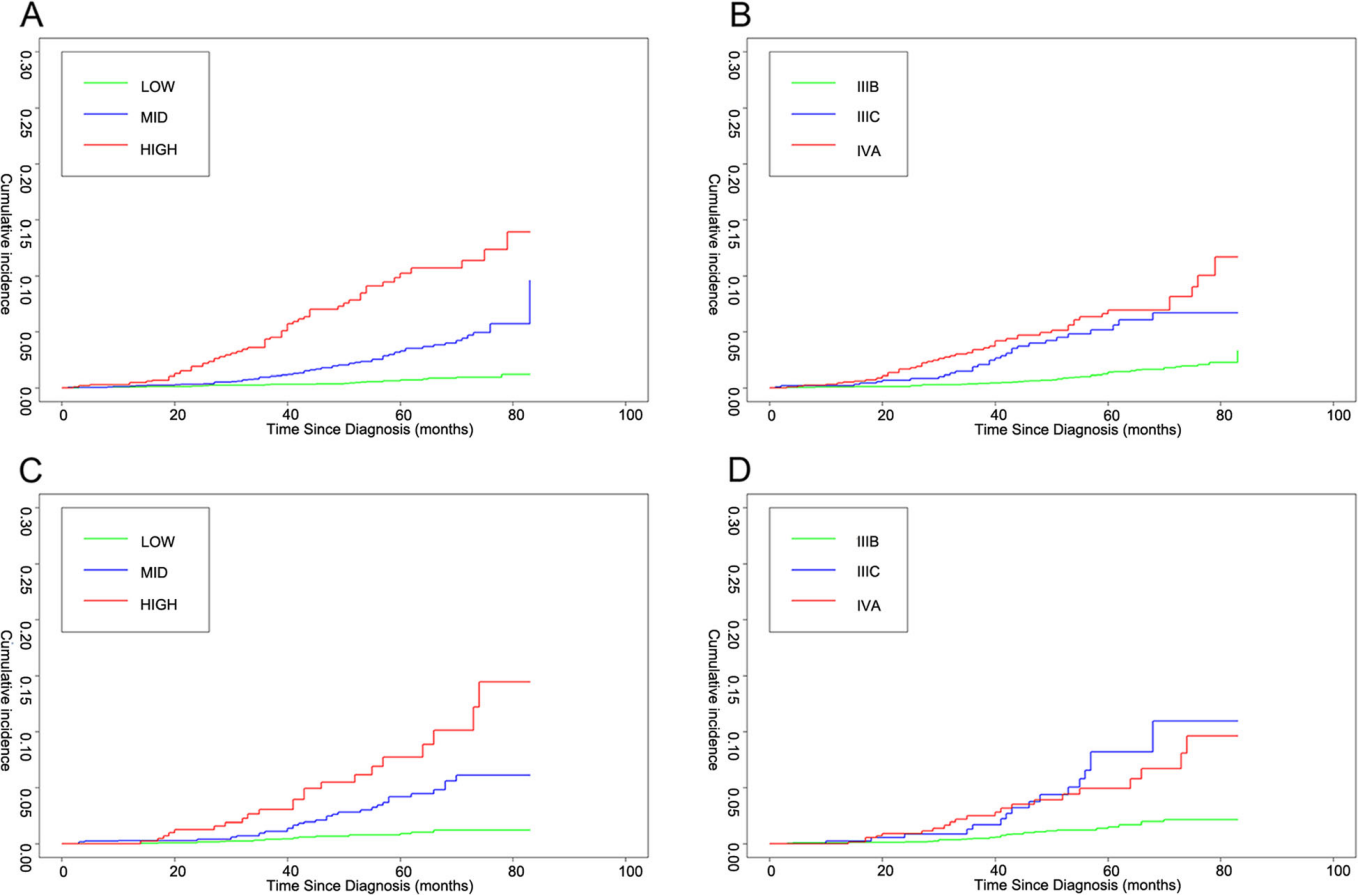
Cumulative incidence function (CIF) curves of different risk stratification systems for cancer specific mortality (CSM) in locally advanced prostate cancer patients after undergoing surgery. Nomogram risk stratification and AJCC staging system were used in the training cohort (**a**, **b**) and validation cohort (**c**, **d**)

The AJCC staging could divide the whole eligible patients in current study into 3 groups including IIIB, IIIC, and IVA. Relatively, as mentioned above, the risk groups based on the nomogram included low-risk group, middle-risk group, high-risk group. For risk groups based on the nomogram, the degree of separation of CIF curves of CSM between groups was more obvious than AJCC staging system in both training cohort and validation cohort (Fig. 4). The CIF curves still demonstrated the new stratification of risk groups based on the nomogram had better prognostic discrimination than AJCC staging system.

## Discussion

Using a large cohort of 14,185 patients from the SEER database, we successfully used 3 independent preoperative prognostic factors to established a nomogram for predicting 5-year CSM for patients with locally advanced PCa. To our knowledge, this is the first preoperative predictive nomogram and nomogram-based risk group stratification built for patients with locally advanced PCa. At the same time, the patient samples are the latest relatively (2010–2016), which is conducive to reducing the impact of defects in pathology, surgery and laboratory testing techniques on the results. This nomogram and nomogram-based risk group stratification is not inferior or even better than the current AJCC staging system in terms of discrimination, and is more quantitative and intuitive, which is convenient for clinicians to use.

Nomograms use a variety of biological and clinical variables to graphically depict the probability that a clinical event may occur for each individual [14]. One of their unique abilities is to estimate individualized risk based on patient and disease characteristics in a graphical and user-friendly form. Nomograms can include many continuous variables and key factors of disease into the prognosis, and taking weights of each variable into account, to make predictive models more practical [14, 15]. Compared with the current prognostic prediction system like AJCC staging system, the nomogram showed advantages in many studies on different cancers [16, 17]. They can also establish individualized risk stratification and help clinicians identify suitable patients for optimal managements, and good results had been obtained in many studies of different tumor types [18–20].

In our study, the statistical analysis highlighted 3 preoperative prognostic factors including clinical N stage, GS, and % positive core. Among them, GS had the greatest contribution to 5-year cancer-specific death (GS shared the most proportion of the nomogram total risk score.). GS is one of the most universal prognostic factors for PCa. International Society of Urological Pathology (ISUP) proposed in 2014, GS can be divided into five groups (2–6, 7(3 + 4), 7(4 + 3), 8, ≥9) according to the differences of prognosis [21]. In our study, the classification of GS also referred to the ISUP grading system, and the risk trend shown by the nomogram was consistent with this grading system. Some previous studies had shown the relationship between the prognosis of PCa and GS [6, 22, 23]. These studies had confirmed the increased possibility of adverse clinical events including death and biochemical recurrence in PCa patients with increasing GS. But these studies did not show what the weight of GS compared to other variables on affecting patients was. % positive core showed the obvious impact on CSS for patients in the current study, only inferior to GS. % positive core refers to the proportion of positive needles in the needle biopsy to the total number of needles. Many studies had revealed the prognostic value of % positive core for PCa [6, 22, 24, 25]. In our nomogram, the risk score of group 75–100% increased significantly from 7 (group 50–75%) to 30, suggesting that 75–100% positive core can more significantly affect the patient’s prognosis. This finding was similar to the results of a study of 195 patients with high-risk PCa who undergoing RP. In that study, 70% positive was shown to have significant prognostic discrimination [24]. From the nomogram, regional lymph node metastasis (N1) was another important independent prognostic factor for CSS of locally advanced PCa patients. Previous studies had demonstrated that lymph node metastasis in most patients with PCa was associated with progressive disease, which was important for the prognosis of patients [26, 27]. A retrospective study of 229 patients indicated that the 5-year disease-free survival of rate of patients with regional lymph node metastasis could be decreased from 85% to approximately 50% [28].

Relatively, the 8th AJCC staging system is currently a general PCa risk stratification system, not aim at specific disease stages and treatment therapies [29]. We compared our nomogram risk group stratification with AJCC staging system, and the results showed that our stratification had advantages in C-index (0.779 versus 0.763 for training cohort, and 0.773 versus 0.745 for validation cohort). At the same time, the nomogram risk stratification’s CIF curve showed a better separation trend in both the training cohort and the validation cohort. There may be several reasons. First, our nomogram risk stratification considered the effect of % positive core on CSS of locally advanced PCa patients after RP, compared to AJCC staging system. AJCC staging system only took GS and clinical N stage into account, but this study and other studies mentioned above confirmed the importance of % positive core. Second, AJCC staging system was not quantitative, and the difference between the effects of GS and clinical N stage on prognosis was not reflected. In our nomogram risk stratification, we used different risk scores to reflect the weight of different prognostic factors, showing the obvious importance of GS among the three prognostic factors. At the same time, such quantitative and graphical tool is also easy for clinicians to use as manuals. In addition, the patients included in our study were more targeted than those in AJCC staging system after filtered by locally advanced PCa and RP. By classifying the prognosis of each patient after surgery, clinicians could better choose whether to perform surgery for each patient.

The main significance of the risk stratification proposed by us is to reveal the potential value of the three prognostic factors for the selection of locally advanced patients to receive RP and the influence weight of each factor on the prognosis of these patients. According to the latest guidelines, radical treatments including RP and RT are still the first-line treatments for locally advanced PCa. Only those patients who are unwilling or unable to receive any form of local treatment, or whose PSA growth is fast, or whose tumor differentiation is poor, are offered androgen deprivation therapy (ADT) monotherapy. The guidelines suggest that RP should be used in highly selective locally advanced PCa patients [30]. But at present, there is no high-level evidence on the appropriate selection criteria of RP or RT and the survival benefits comparison between RP and RT for locally advanced PCa patients. In our study, the risk stratification proposed by the nomogram provided a reference for the selection criteria of RP. Considering the difference of postoperative CSS, patients in high-risk group may be not suitable for RP, while patients in low-risk group may be the appropriate population for RP. For those who are not suitable for RP, the RT + ADT therapy may bring more survival benefits. In fact, previous studies have shown that RT + ADT therapy can bring significant benefits to locally advanced PCa patients with pN+ [31, 32]. However, they didn’t compare RP and RT directly. More clinical studies are needed to explore the most reasonable criteria for the selection of locally advanced PCa patients receiving RP or RT, especially the double arm randomized controlled studies of RP and RT, such as the SPCG-15 currently in progress [33].

There are still several limitations to our study. First, our research is constructed by retrospective data. Therefore, there may be some undetected potential bias factors in the study. Second, due to the SEER database’s limitations, we lack functional data and disease progression data for further research. Third, we also lack additional independent external validation sets, and this is our important work goal in the future.

## Conclusions

By analyzing a large cohort of 14,185 patients, we successfully revealed 3 independent preoperative prognostic factors for CSS of locally advanced PCa patients after RP. Using these 3 factors, we constructed and validated a preoperative prognostic nomogram that could predict the probability of CSM at 5 years of this type of patients after undergoing RP. Based on the nomogram, a risk stratification system was established to help clinicians better identify patients suitable for surgery.

## Data Availability

All analyzed data are publicly available at the SEER website (http://www.seer.cancer.gov), and should be requested under the approval of the SEER Program administration.

## Abbreviations

ADT: Androgen deprivation therapy
AJCC: American Joint Committee on Cancer
CI: Confidence interval
CIF: Cumulative incidence function
CSM: Cancer-specific mortality
CSS: Cancer-specific survival
GS: Gleason score
OS: Overall survival
PCa: Prostate cancer
PSA: Prostate specific antigen
RFS: Recurrence-free survival
RP: Radical prostatectomy
SEER: The Surveillance, Epidemiology, and End Results
